# Salinity Effects on Gene Expression, Morphological, and Physio-Biochemical Responses of *Stevia rebaudiana* Bertoni In Vitro

**DOI:** 10.3390/plants10040820

**Published:** 2021-04-20

**Authors:** Clara R. Azzam, Sudad K. Al-Taweel, Ranya M. Abdel-Aziz, Karim M. Rabea, Alaa I. B. Abou-Sreea, Mostafa M. Rady, Esmat F. Ali

**Affiliations:** 1 Cell Research Department, Field Crops Research Institute, Agricultural Research Center, Giza 12619, Egypt; clara.azzam@arc.sci.eg; 2 Medical and Aromatic Plants Research Unit, College of Agriculture Engineering Sciences, University of Baghdad, Al-Jadiriya, Baghdad 10071, Iraq; sudad.altaweel@coagri.uobaghdad.edu.iq; 3 Sugar Crops Research Institute, Agriculture Research Center, Ministry of Agriculture, Giza 12619, Egypt; snowrosa114@gmail.com; 4 Horticulture and Landscape Design Department, College of Agriculture Engineering Sciences, University of Baghdad, Al-Jadiriya, Baghdad 10071, Iraq; Kareemmeayan@gmail.com; 5 Department of Horticulture, Faculty of Agriculture, Fayoum University, Fayoum 63513, Egypt; aib00@fayoum.edu.eg; 6 Botany Department, Faculty of Agriculture, Fayoum University, Fayoum 63514, Egypt; 7 Department of Biology, College of Science, Taif University, P.O. Box 11099, Taif 21944, Saudi Arabia; a.esmat@tu.edu.sa

**Keywords:** stevia, callus induction, salt tolerance, regeneration, total chlorophyll, proline, peroxidase, polyphenol oxidase, malate dehydrogenase

## Abstract

*Stevia rebaudiana* Bertoni is a little bush, which is cultivated on a large scale in many countries for medicinal purposes and used as a natural sweetener in food products. The present work aims to conduct a protocol for stevia propagation in vitro to produce and introduce *Stevia rebaudiana* plants as a new sweetener crop to Egyptian agriculture. To efficiently maximize its propagation, it is important to study the influence of stress factors on the growth and development of *Stevia rebaudiana* grown in vitro. Two stevia varieties were investigated (Sugar High A3 and Spanti) against salt stress. Leaves were used as the source of explants for callus initiation, regeneration, multiplication and rooting. Some stress-related traits, i.e., photosynthetic pigments, proline contents, and enzyme activity for peroxidase (POD), polyphenol oxidase (PPO), and malate dehydrogenase (MDH) were studied. Murashig and Skoog (MS) medium was supplemented with four NaCl concentrations: 500, 1000, 2000, and 3000 mgL^−1^, while a salt-free medium was used as the control. The data revealed that salinity negatively affected all studied characters: the number of surviving calli, regeneration%, shoot length, the number of multiple shoots, number of leaf plantlets^−1^, number of root plantlets^−1^, and root length. The data also revealed that Sugar High A3 is more tolerant than Spanti. The total chlorophyll content decreased gradually with increasing NaCl concentration. However, the opposite was true for proline content. Isozyme’s fractionation exhibited high levels of variability among the two varieties. Various biochemical parameters associated with salt tolerance were detected in POD. Namely, POD4, POD6, POD 9 at an *Rf* of 0.34, 0.57, and 0.91 in the Sugar High A3 variety under high salt concentration conditions, as well as POD 10 at an *Rf* of 0.98 in both varieties under high salt concentrations. In addition, the overexpression of POD 5 and POD 10 at *Rf* 0.52 and 0.83 was found in both varieties at high NaCl concentrations. Biochemical parameters associated with salt tolerance were detected in PPO (PPO1, PPO2 and PPO4 at an *Rf* of 0.38, 0.42 and 0.62 in the Sugar High A3 variety under high salt concentrations) and MDH (MDH 3 at an *Rf* of 0.40 in both varieties at high salt concentrations). Therefore, these could be considered as important biochemical markers associated with salt tolerance and could be applied in stevia breeding programs (marker-assisted selection). This investigation recommends stevia variety Sugar High A3 to be cultivated under salt conditions.

## 1. Introduction

*Stevia rebaudiana* Bertoni is a little bush, a member of the *Asteraceae* (composite) family, and an important medicinal plant native to northeastern Paraguay and southern Brazil. *Stevia rebaudiana* is now being cultivated on a large scale in many other countries including Japan, Taiwan, Korea, Thailand, and Indonesia for medicinal purposes and is used as a natural sweetener in food products [1]. In Egypt, there is great interest in the strategic plan for the cultivation of a new crop to be used as a source of natural sweeteners. Its cultivation could be used as an alternative for the new land reclamation projects to reduce the sugar demands of the Egyptian markets and generate income for the growers [2]. The nine types of steviol glycosides found in stevia are stevioside, rebaudioside A, B, C, D, E, and F, dulcoside A, and steviolbioside. The glycosides are non-mutagenic, non-toxic and low in calories, contrary to conventional sugar substitutes, such as sorbitol or xylitol [3].

Salinity is one of the most significant abiotic factors, which negatively influences plant efficiency and the production of many crops, such as peanuts [4,5], wheat [6,7,8,9], alfalfa [10], common bean [11], onion [12], cucumber [13] and soybean [14]. Around 19.5% of irrigated agricultural land is considered to be saline [15]. Even though minerals are fundamental for plants, their excessive abundance in the soil is harmful to plants. Plants subjected to an excess of harmful ions or a physiological water deficit due to salinity are threatened by oxidative stress linked to the overproduction of ROS (reactive oxygen species), which are violent enemies that damage nucleic acids, proteins, and lipids [16]. Oxidative stress is viewed as one of the most significant damaging factors in plant cells exposed to salt stress [17,18]. Plants grown in fields with high levels of salinity are affected by osmotic stress, and therefore, they encounter a great deal of negative effects at the morphological, physiological and biochemical levels [19]. It is difficult to detach systemic from cellular salinity tolerance mechanisms because of the structural complexity of the entire plant [20,21]. Therefore, it has become important for us to determine the changes in these characteristics.

For stevia, germination and establishment from seeds are often poor and sometimes unsuccessful, which hinders its agricultural application. Propagation by seeds does not allow for the production of a homogenous population, resulting in variability in the level of sweetness and composition, affect their quality, quantity and growth. Stevia plants are mostly spread by vegetative propagation via stem cutting and by micropropagation using tissue culture practices. Therefore, studies have suggested that tissue culture is the best option for the multiplication of stevia plants [22,23] as plants can recover from dislocated callus tissues [24,25]. Salt stress results in stevia demonstrating alterations in biomass, antioxidant enzyme activities and osmolytes, mineral content, secondary metabolites, and productivity [26]. Plant tissue culture presents various opportunities for researchers to study the unique and complex responses of plants to environmental stresses. Plant tissue culture is significant in improving salt tolerance in plants, which indicates a prolonged period [27,28]. Recently, tissue culture strategies have been utilized as a valuable instrument to clarify the mechanisms engaged with salt tolerance by utilizing chosen salt-lenient cell lines in vitro; consequently, producing salt-lenient plants [29,30,31]. Stevia plantlet growth characters decrease as salinity stress levels increase [32]. Zeng et al. [33] reported that *S. rebaudiana* is mildly tolerant to salt stress. Increasing the NaCl concentration (25, 50, 75, and 100 mM) in the medium, decreases the shoot number and shoot length of stevia significantly [34]. However, the rate of proline accumulation has been shown to increases with decreases in protein content as a consequence of the increasing salt concentration. In this respect, the chlorophyll content was observed to decrease—as compared to sugars, proline, and phenols—with increasing salt concentration. Moreover, the results showed that osmotic stress significantly reduced the growth and yield components of *S. rebaudiana* Bertoni which is in agreement with Rathore et al. [35].

Biochemical methods, especially isozyme studies, have provided valuable tools for breeders. Isozymes can serve as unique molecular genetic markers for biochemical characterization of genotypes [36]. Antioxidant enzymes are the main compounds to protect plants from the cytotoxic effects of reactive oxygen species (ROS) in stress conditions. NaCl stress can catalyze or suppress the expression of various antioxidant enzyme isoforms and related stress enzyme induction that is potentially associated with the ROS level, which causes cell damage due to oxidative stress [37]. In in vitro studies, increasing the salinity induced three types of effects: (1) an increase in peroxidase activity; (2) a decrease in catalase activity; (3) inhibition of malate dehydrogenase activity [38]. The expression of various antioxidant enzymes such as peroxidase (POD) and polyphenol oxidase (PPO) has a positive correlation with high levels of abiotic stress tolerance. Studies such as these demonstrate the important relationship between antioxidant activity and salt tolerance development, which may cause some changes in gene expression [39]. Salinity tolerance is unlikely to be determined by a single gene or gene product [40]. The present work was conducted to display the protocol for stevia propagation through tissue culture techniques to produce and introduce *Stevia rebaudiana* plants as a new sweetener crop in Egyptian agriculture. To efficiently maximize plant propagation, it is important to study the influence of stress factors on the growth and development of *Stevia rebaudiana* grown in vitro. Therefore, this investigation was carried out to study the effect of salt stress, using different concentrations of sodium chloride (NaCl), on calli and their recovery, morphological characteristics, proline accumulation, total chlorophyll, and the biochemical activity of two stevia varieties—Sugar High A3 and Spanti—in vitro.

## 2. Results

### 2.1. Effect of NaCl on Morphological Responses of Stevia Rebaudiana Bertoni In Vitro

Analysis of variance revealed highly significant differences (*p* ≤ 0.05) among the two stevia varieties viz. Sugar High A3 and Spanti as regards survival callus%, germination%, plantlet length, number of leaf plantlets^−1^, number of multiple shoots, root length, and number of root plantlets^−1^ traits and responses against five NaCl concentrations: 0, 500, 1000, 2000, and 3000 mgL^−1^, as well as their interaction (Table 1).

Salinity greatly affected the survival percentage of survival callus% and regeneration% in both stevia varieties (Sugar High A3 and Spanti). The percentage of survival callus and its ability to renew growth was affected under the stress conditions of different NaCl salinity levels. The mean regeneration percentage reached the maximum number with free-salt control and decreased gradually with increasing NaCl concentrations until it was the lowest at 3000 mg L^−1^. Salinity was more harmful to the Spanti variety (45.01 and 25.30%, respectively, for survival callus and regeneration%) than the Sugar High A3 variety (52.13 and 35.90%, respectively, for survival callus% and regeneration%). Interactions between salinity and varieties showed that the highest salinity concentration (3000 mg L^−1^ NaCl) induced the lowest percentage of survival callus% and regeneration%, especially for the Spanti variety (32.65, and 18.09%, respectively) compared with the salt-free control (62.0, and 34.93%, respectively), as shown in Table 1.

A significant difference was detected between the two stevia varieties. It was feasible that salinity did not reduce the average length of the plantlet in the Spanti variety. The Sugar High A3 variety gave a higher value (4.76 cm) than Spanti (4.08 cm). The salinity level of 500 mg L^−1^ NaCl gave the highest value of plantlet length (5.66 cm) in the Sugar High A3 variety.

**Figure 1:**
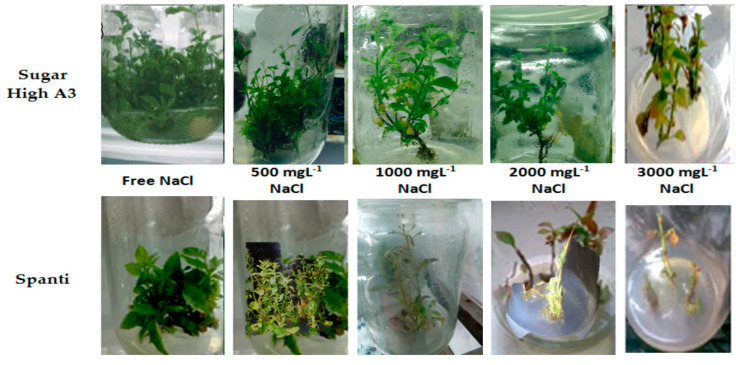
Effects of different NaCl concentrations (0, 500, 1000, 2000, and 3000 mgL^−1^) growth and development of stevia varieties (Sugar High A3 and Spanti) in vitro.

Additionally, the number of multiple shoots and the number of leaves per plant were affected by the rising salinity level in the medium (Table 1 and Figure 1). The highest significant number of multiple shoots and the number of leaves per plant of the Sugar High A3 was achieved at the salt-free control (15.60, and 9.61), while the lowest significant means were 10.23, and 6.11, respectively, with 3000 mg L^−1^ NaCl. Salinity has more of an effect on the Spanti than the Sugar High A3 variety.

**Figure 2:**
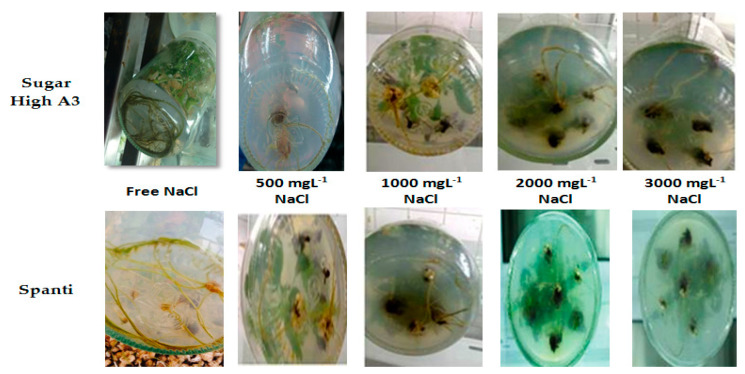
Effects of different NaCl concentrations (0, 500, 1000, 2000, and 3000 mgL^−1^) on root development of stevia varieties (Sugar High A3 and Spanti) in vitro.

Salinity had an apparent significant effect on the number of roots per plantlet and root length, as shown in Table 1 and Figure 2. The highest significant values were achieved with the NaCl-free medium for the two varieties, whereas the Sugar High A3 variety recorded 6.24, and 6.66, respectively, for the number of roots per plantlet and root length, while the lowest significant value was recorded at 3000 mgL^−1^ (5.01, and 6.06, respectively). Meanwhile, the lowest values in the Spanti stevia variety were recorded with NaCl concentration of 2000 mgL^−1^, as shown in Table 1 and Figure 2.

### 2.2. Effect of NaCl on Physiological Responses of Stevia Rebaudiana Bertoni In Vitro

**Figure 3:**
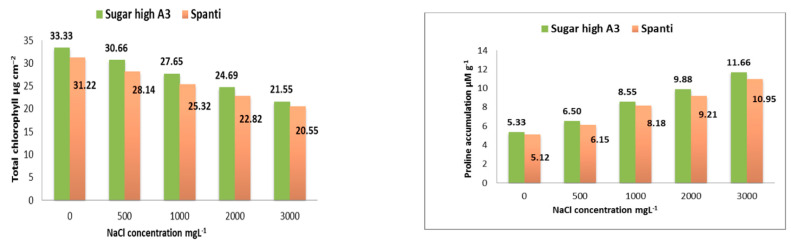
Effects of different NaCl concentrations (0, 500, 1000, 2000, 3000 mgL^−1^) on proline (μM g^−1^) accumulation and total chlorophyll (μg cm^−2^) of stevia varieties (Sugar High A3 and Spanti) in vitro.

The effects of salinity stress on the total chlorophyll concentration in Sugar High A3 and Spanti stevia varieties are shown in Figure 3. The photosynthetic pigments (total chlorophyll concentration) decreased in parallel with increases in the salt concentration in both varieties in vitro. There were differences between the salt-free treatment and all NaCl concentrations: 500, 1000, 2000, and 3000 mgL^−1^. At the highest salt concentration of 3000 mgL^−1^ NaCl, the total chlorophyll concentration was 21.55 and 20.55 µg cm^−2^, compared with the control (salt-free medium), which recorded 33.33 and 31.22 µg cm^−2^ in Sugar High A3 and Spanti varieties, respectively. The reduction rates in comparison with the salt-free treatment (0 NaCl) were −35.34 and −34.17%, respectively, in the Sugar High A3 and Spanti varieties.

The effects of salinity stress on proline accumulation in the Sugar High A3 and Spanti stevia varieties are shown in Figure 3. As the NaCl concentration was increased in the MS medium, in parallel, the accumulation of proline content increased. In terms of the proline average across the salinity level, there was a significant difference (*p* ≤ 0.05) in the content of proline between the two stevia varieties. At the highest concentration of 3000 mg L^−1^ of NaCl, the proline concentration was increased by 118.76 and 113.87%, respectively, in Sugar High A3 and Spanti stevia varieties compared with the control (0 NaCl) that was 5.33 and 5.12 µM g^−1^, respectively.

### 2.3. Effect of NaCl on Gene Expression and Biochemical Response of Stevia Rebaudiana Bertoni In Vitro

**Figure 4:**
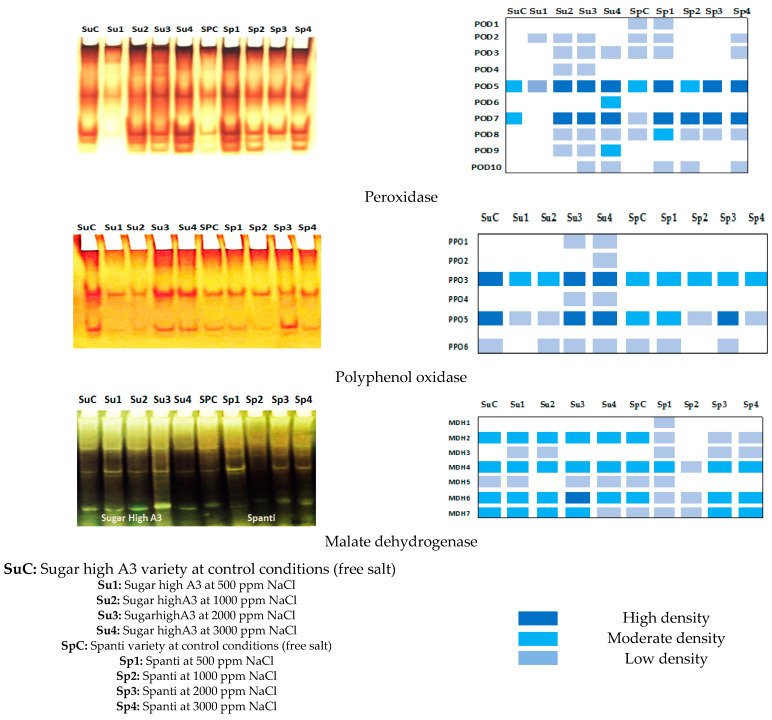
Zymogram and ideogram analysis of peroxidase, polyphenol oxidase, and malate dehydrogenase isozymes banding patterns from leaves of stevia varieties (Sugar High A3 and Spanti) grown in different NaCl concentrations (0, 500, 1000, 2000, and 3000 mgL^−1^) in vitro.

In the present study, an attempt was made to assay the variation of number and activity of three isozymes patterns: peroxidase, polyphenol oxidase, and malate dehydrogenase in the leaf crude extract of two stevia varieties: Sugar High A3 and Spanti, grown under control (salt-free medium) and four NaCl concentrations. Variations in the number and activity of bands are shown in Figure 4 and Table 2, Table 3 and Table 4 for peroxidase, polyphenol oxidase, and malate dehydrogenase, respectively.

Genetic polymorphisms in the peroxidase isozyme were assessed in the Sugar High A3 and Spanti stevia varieties grown under control and different NaCl concentration conditions to illustrate differences in the patterns. Zymograms and ideogram analysis (Figure 4) and densitometric analyses (Table 2) show the electrophoretic profiles of the peroxidase enzyme system in stevia plants. The peroxidase isozyme exhibited a wide range of variability among the different salt concentrations. Native PAGE showed ten POD isozyme groups: POD1, POD2, POD3, POD4, POD5, POD6, POD7, POD8, POD9, and POD10 at *Rf* (0.18, 0.23, 0.29, 0.34, 0.52, 0.57, 0.83, 0.87, 0.91 and 0.98). A total number of 52 bands were detected in the present materials. The untreated variety showed six POD bands in the case of the Spanti variety, while two bands appeared as total POD bands in the Sugar High A3 variety, which was the lowest number of peroxidase isozyme bands. Salinity-stressed plantlets showed highly overexpressed POD, which produced eight bands including six faint bands at *Rf* (0.23, 0.29, 0.34, 0.57, 0.87, 0.91, and 0.98), and two highly dense bands at *Rf* (0.52 and 0.83) in the Sugar high A3 variety. Conversely, in the Spanti variety, POD recorded seven bands including five faint bands at *Rf* (0.18, 0.23, 0.29, 0.87, and 0.98), and two highly dense bands at *Rf* (0.52 and 0.83). Peroxidase isozymes showed high polymorphism values (90%) based on the zymogram number, relative mobility (*Rf*), and optical intensity. The band at *Rf* of 0.52 could be considered as a common band with relative differences in optical intensity. Peroxidase isozyme intensity was increased in response to stress, with the highest magnitude of induction being an adaptive strategy under stress.

Genetic polymorphisms in the polyphenol oxidase enzyme system were studied in stevia plants of the two varieties: Sugar High A3 and Spanti, grown under control and different NaCl concentrations conditions, to illustrate differences in patterns. Zymograms, ideogram analysis (Figure 4) and densitometric analyses (Table 3) show the electrophoretic profiles of the polyphenol oxidase enzyme system in stevia plants. The polyphenol oxidase isozyme exhibited a wide range of variability among the different salt concentrations. Polyphenol oxidase isozymes showed high values of polymorphisms (75%) based on the zymogram number, relative mobility (*Rf*), and optical intensity. Sugar High A3 exhibited the highest gene expression value for PPO compared with the Spanti variety. Native PAGE showed six PPO isozyme groups: PPO1, PPO2, PPO3, PPO4, PPO5, and PPO6 at *Rf* of 0.38, 0.42, 0.58, 0.62, 0.55, and 0.88. A total number of 32 bands were detected across stevia varieties and NaCl concentrations. PPO3 and PPO5 groups at an *Rf* of 0.58, and 0.85 could be considered as common bands with relative differences in optical intensity. Three PPO groups appeared in both varieties grown under the salt-free medium (PPO3, PPO5, and PPO6), as well as salt-stressed Spanti variety, while salinity-stressed plantlets of the Sugar High A3 variety showed highly overexpressed PPO, leading to the production of six bands, three of them were newly expressed at *Rf* 0.38, 0.42, and 0.62 at the high salt concentration of 2000 and 3000 mgL^−1^ NaCl. Polyphenol oxidase isozyme intensity was increased in response to stress with the highest magnitude of induction as an adaptive strategy under stress.

The electrophoretic profiles of the malate dehydrogenase (MDH) isozyme showed that the isozyme activities were affected by NaCl treatment. The effects of NaCl were more obvious in terms of the activity of constitutive enzyme pools. In the present study, we observed differential MDH isozyme activities among two stevia varieties: Sugar High A3 and Spanti, grown under control and different NaCl concentrations (Figure 4) and Table 4. Zymogram analysis, ideogram analysis (Figure 4) and densitometric analyses (Table 4) show the electrophoretic profiles of the MDH system in stevia plants. The MDH isozyme exhibited a wide range of variability among the different salt concentrations. 

MDH isozymes showed moderate polymorphism values (57%) based on the zymogram number, relative mobility (*Rf*), and optical intensity. The highest values of gene expression for MDH were higher in the Spanti variety than in the Sugar High A3 variety. Native PAGE showed seven MDH isozyme groups: MDH 1, MDH 2, MDH 3, MDH 4, MDH 5, MDH 6 and MDH 7 at *Rf* 0.29, 0.33, 0.40, 0.52, 0.55, 0.078 and 0.83. A total number of 51 bands were detected across stevia varieties and NaCl concentrations. MDH 4, MDH 6, and MDH 7 groups at *Rf* of 0.52, 0.78, and 0.83 could be considered as common bands across the two stevia varieties and salt concentrations with relative differences in optical intensity. Five MDH groups appeared in both varieties grown under the salt-free medium (MDH 2, MDH 4, MDH 5, MDH 6, and MDH 7), while the salt-stressed Spanti and Sugar High A3 varieties expressed a new MDH group at *Rf* 0.40, in addition to MDH 1, that was newly expressed in the Spanti variety grown under the salt concentration of 500 mgL^−1^ NaCl at *Rf* 0.29. Malate dehydrogenase isozyme intensity was increased in response to stress with the highest magnitude of induction as an adaptive strategy under stress.

## 3. Discussion

Salinity stress is considered one of the most critical challenges facing countries, especially Egypt. Growing knowledge of environmental problems makes it important to seek alternatives that are easy to use and feasible to overcome the harmful impacts of salinity on plants [41]. In the present study, plant growth parameters such as survival callus%, germination%, and all morphological traits in regard to plantlet length, number of leaf plantlets^−1^, number of multiple shoots, root length, and number of root plantlets^−1^ were inhibited by NaCl stress. The physiological parameters of callus formation and shoot formation were significantly declined in both stevia varieties: Sugar High A3 and Spanti, when the MS medium was supplemented with up to 500 mg L^−1^ NaCl concentration. Increasing NaCl concentration during the formation of regenerants was observed. The best growth was obtained in the case of the control during root formation (Table 1). The finding of this study leads to the conclusion that significantly less growth was observed after exposure to rising salt stress, while the control treatment produced the best results concerning shoot number, shoot length (cm), number of leaves, number of multiple shoots, leaf number, root number, root length (cm), % survival callus, and % regeneration. The most probable reason for growth reduction in the case of root formation is the change in metabolic activities resulting in a reduction in cell division, elongation, and differentiation of roots [32]. Plant height decreased under NaCl concentrations. As with increasing salinity, the values of all studied morphological traits decreased, i.e., shoot length of stevia grown in vitro in different concentrations of NaCl (0, 20, 40, 60, and 80 mM) [42]. The decrease in plant height due to treatment with NaCl could be attributed to the inhibition of growth which negatively affected the growth hormones due to salinity, as well as photosynthetic pigments and enzyme activity [43,44]. The soil osmotic effect and limited uptake of water by the plant represent the earliest consequences of NaCl stress [45]. In response to this effect, the stevia plant may display a decrease in evapotranspiration and stomatal conductance during the stress period, as described in other plant species [46,47]. The response of the plant to salt stress varied at diverse developmental phases. Increasing salinity throughout plant development may interrupt vegetative growth, germination, and the formation of thinner roots. Salinization can constrain both cell separation and cell expansion in growing tissues of stems, roots, and leaves. The amount of Na^+^ absorption was almost equally divided in both roots and shoot organs [48,49,50,51]. Accumulation of Na^+^ occurs in plants growing under salinity stress and leaves are more vulnerable than roots to Na^+^, simply because Na^+^ accumulates to higher concentrations in shoots than in roots [52]. A significant decrease in root number and root length of stevia were observed in vitro by increasing the NaCl and mannitol concentration (25, 50, 75, and 100 mM), as reported by Pandey and Chikara [34]. Stevia is a moderate salinity tolerant plant, as described previously by Zeng et al. [33] and Cantabella et al. [53], which is in agreement with the findings of this work.

The current results mentioned that the photosynthetic pigments (total chlorophyll concentration) decreased in parallel with salt concentration increases in both varieties in vitro (Figure 3). The salinity-tolerant ability of plants is controlled by many physiological processes, among them, photoassimilate is inhibited under salt stress and the degree of reduction in photoassimilate is positively proportional to stress strength [54]. Chlorophyll (Chl) and carotenoids contents have been proven to be important indicators of the severity of osmotic stress affecting plants [55]. The decrease in total Chl under NaCl stress is due to the increase in activity of chlorophyllase, promoting its degradation [56]. In the present study, the results are in harmony with the negative effect of NaCl stress on the total Chl (Figure 3). High salinity (NaCl) also considerably affects the process of photosynthesis in most plants by altering the ultrastructure of the organelles and pigment concentrations [57]. Likewise, Zeng et al. [33] and Cantabella et al. [53], in a previous study, described a significant decrease in chlorophyll content in stevia plants. The change in chlorophyll concentration, owing to salinity, is the most deceptive biochemical response [58, 59]. In salt-stressed plants, chlorophyll concentration was expressively reduced, relying on NaCl concentration [60]. This drift has been informed before by Khawale et al. [61]. El-Sabrout [62] claimed that Na^+^, Cl^−^, and free proline contents presented a tendency of positive responses to salinity treatment. The depressive impact of salt stress on chlorophyll biosynthesis might be because of the formation of proteolytic enzymes such as chlorophyllase, which is accountable for chlorophyll degradation and harming the photosynthetic device. The decrease in total chlorophyll under NaCl stress is due to the increase in activity of chlorophyllase, promoting its degradation [56]. In the present study, our results are in harmony with the negative effect of NaCl stress on the total chlorophyll content. High salinity (NaCl) also considerably affects the process of photosynthesis in most plants by altering the ultrastructure of the organelles and pigments concentration [57]. Likewise, Zeng et al. [33] and Cantabella et al. [53] previously described a significant decrease in the chlorophyll content in stevia plants. The reduction in photosynthetic pigments at higher salinities, i.e., total chlorophyll in this experiment, may be due to the accumulation of a high amount of Na^+^ in the leaf, which disrupts the ultra-structure of the chloroplast and breaks down the chlorophyll. Under salinity stress conditions, chlorophyll degradation occurs due to enhanced activity of the chlorophyllase enzyme [63]. However, some researchers have explained that Cl^–^ toxicity is the primary reason for the degradation of chlorophyll in plants [64].

The results of this study confirmed that as NaCl concentrations were increased in the MS medium, in parallel, the accumulation of proline content increased. At the highest concentration of 3000 mg L^−1^ of NaCl, the proline concentration was increased by 118.76 and 113.87%, respectively, in Sugar High A3 and Spanti stevia varieties, as compared with the control. The high value of proline with NaCl at 3000 mg L^−1^ could be due to the fact that the stevia plant faces osmotic stress under salinity conditions and proline is produced for osmotic adjustment. However, the catabolism of proline is enhanced during recovery [65] and during this phase, proline regulates cell proliferation, cell death, and the expression of stress recovery genes [66]. Among the studied metabolites, proline was accumulated in the leaves under the effect of salt stress. Proline is an indispensable compound in studies related to osmotic stress, and we observed its accumulation in the leaves of NaCl-treated stevia plantlets, which is in accordance with other published research [67]. Plant cells have the potential to accumulate proline speedily and degrade it quickly when needed [67]. However, highlighting the role of proline in plant adaptation is not always reliable because in many studies, no elevation of proline was observed in plants under osmotic stress [55]. Additionally, some studies reported that proline accumulation was the most pronounced under salt-induced osmotic stress: the highest proline accumulation was detected in the leaves of NaCl-treated plants [55]. The decrease or increase in proline induction due to iso-osmotic stress (salt) indicated the suitability or tolerance of the species to abiotic stress. Thus, when proline production is increased, this indicates that the genotype can afford salt stress and it is tolerant to iso-osmotic stress, which indicates that *Stevia rebaudiana* Bertoni may be tolerant to salt stress. Disturbances to plant metabolism induced by abiotic treatments generally affect the various metabolic pools of iso-osmotic stressed plants. These changes in the contents of the various metabolites under salt treatments may indicate an enhancement or retardation of the synthesis, accumulation, or consumption of these cellular metabolites. Proline, which frequently accumulates in stressed cells more than any other free amino acid, was always correlated with the stress to which the plant cell is subjected. However, the values of these contents varied according to the degree of stress, the plant species tested and the organ analyzed. These results are in accordance with those obtained by other authors, i.e., the accumulation of proline is frequently reported for most of the plant cells and tissues exposed to stress [68]. The change in proline content has been correlated with its capacity to tolerate and adapt to salinity conditions. Several research articles have connected the accumulation of free proline to salt stress, for instance, the jojoba plant [69].

Biochemical methods of investigation, especially isozyme studies, have provided valuable tools for breeders. Isozymes can serve as unique molecular genetic markers for the biochemical characterization of genotypes [36]. The results of this study showed that peroxidase isozymes produced high polymorphism values (90%) based on the zymogram number, relative mobility (*Rf*), and optical intensity. Peroxidase isozyme intensity was increased in response to stress, with the highest magnitude of induction being an adaptive strategy under stress. An increase in peroxidase activity probably represents the induced protective reaction delaying senescence. A previous study reported an increase in activity of the antioxidant enzymes and the transcript levels of their encoding genes under salt stress in seedlings of *Limonium sinense* Kuntze [70]. To improve salt tolerance, various tools were used, such as natural variations, transgenic plants, novel gene introduction, and/or alteration of gene expression [71]. The over-expression of specific stress response genes in plants capable surviving in each stress environment is evoked as a common adaptive mechanism. To conserve plant cells from oxidative damage induced by abiotic stresses, such as salt stress, the organization of several genes encoding antioxidant enzymes is increased. Therefore, analysis of transcriptional levels of antioxidant defense genes can provide insight into the levels required for a salt stress response in plants. To protect plant cells from oxidative damage caused by abiotic stresses, such as salt stress, many genes encoding antioxidant enzymes are regulated. Therefore, analyses of the transcriptional levels of antioxidant defense genes could take into account their payoff in the salt stress response in plants. Plant peroxidases have been used as biochemical markers for various types of biotic and abiotic stresses due to their role in very important physiological processes, such as the control of growth by lignification, cross-linking of pectins and structural proteins in the cell wall, and the catabolism of auxins.

We know the importance of the peroxidase isoenzyme in catalyzing the reaction that protects the plants, against damage by free radicals. Populations showing low peroxidase activity have shown that this may not adapt them at wider ranges because plants may lose permeability of the membrane and proceed toward the end of life due to the harmful action of free radicals. The lipids of membranes, where peroxidation of unsaturated fatty acids takes place, are the main cellular components that are susceptible to damage by free radicals. Furthermore, the changes in band intensity could be interpreted based on gene duplication or point mutations that lead to the production of shorter and longer polypeptide chains and the alteration of structural genes, which may be due to changes in regulator gene(s) expression [72]. The isozyme results are coherent with Azzam et al. [73] and Abbas et al. [4].

The current research findings showed that polyphenol oxidase isozymes produced high polymorphism values (75%) based on the zymogram number, relative mobility (*Rf*), and optical intensity. The highest values of gene expression for PPO were higher in the Sugar High A3 variety than the Spanti variety. Salinity-stressed plantlets of the Sugar High A3 variety showed more highly overexpressed PPO than the Spanti variety and three new groups expressed at *Rf* 0.38, 0.42, and 0.62 at the high salt concentration of 2000 and 3000 mg L^−1^ NaCl. The polyphenol oxidase isozyme intensity was increased in response to stress with the highest magnitude of induction as an adaptive strategy under stress. An increase in polyphenol oxidase isozyme activity probably represents an induced protective reaction, delaying senescence. Polyphenol oxidase isozyme intensity was increased in response to stress, with the highest magnitude of induction in older leaves and corresponding to abscission zones. This might preferentially facilitate cell death in these tissues, as an adaptive strategy under stress, since it could reduce further water loss and allow for limited nutrients to be partitioned to younger tissues. Furthermore, the changes in band intensity could be interpreted based on gene duplication or point mutation that leads to the production of shorter and longer polypeptide chains and alteration in the structural genes, which may be due to the changes in regulator gene(s) expression [72]. Previous studies have discussed that polyphenols synthesis relies on abiotic factors such as environmental factors [74] and salinity [75,76]. Salinity-stimulated disturbances to plant metabolism could cause an increase in many phenolic compounds, as claimed by Radi et al. [77].

To detect the tolerance or sensitivity of stevia to salinity, enzyme activity, such as biochemical and gene expression, as molecular markers of peroxidase and polyphenol oxidase could be used. The changes in peroxidase and polyphenol oxidase have a positive correlation with high levels of abiotic stress tolerance. On the other hand, some enzyme activation is responsible for protecting plants against oxidative damage. Such investigations have revealed the important relationship between antioxidant activity and salt tolerance, which may be commensurate with its ability to withstand salt stress or because of salt stress, which may cause some change in gene expression.

In the present study, we observed differential MDH isozyme activities among two stevia varieties: Sugar High A3 and Spanti, grown under control and different NaCl concentrations (Figure 4) and (Table 4). MDH isozyme exhibited a wide range of variability among the different salt concentrations. MDH isozymes showed moderate values of polymorphism (57%) based on the zymogram number, relative mobility (*Rf*), and optical intensity. The highest values of gene expression for MDH were higher in the Spanti variety than the Sugar High A3 variety. Salt-stressed Spanti and Sugar High A3 varieties expressed a new MDH group at *Rf* 0.40, in addition to MDH 1 that was newly expressed in the Spanti variety grown under a salt concentration of 500 mg L^−1^ NaCl at *Rf* 0.29. The malate dehydrogenase isozyme intensity was increased in response to stress with the highest magnitude of induction as an adaptive strategy under stress. An increase in MDH activity probably represents an induced protective reaction, delaying senescence.

The malate dehydrogenase isozyme intensity was increased in response to stress, with the highest magnitude of induction in older leaves and corresponding to abscission zones. This might preferentially facilitate cell death in these tissues, as an adaptive strategy under stress, since it could reduce further water loss and allow for limited nutrients to be partitioned to younger tissues. Furthermore, the changes in band intensity could be interpreted based on gene duplication or point mutations that lead to the production of shorter and longer polypeptide chains and the alteration of structural genes, which may be due to changes in regulatory gene expression [72].

The activity of MDH is serving as an objective enzyme test for determining cereal genotypes with high tolerance to salt stress [78, 79]. These results are in agreement with Biruk et al. [80], who found that MDH gave two monomorphic areas, but only some bands were variable between the studied stevia cultivars. Isozymes are the multiple molecular forms of a single enzyme with identical substrate specificity. They vary frequently in their patterns in berseem intact plants as a function of various physiological and developmental stages [73].

## 4. Materials and Methods

This work was carried out successfully in the Tissue Culture Laboratories of the Institute of Sugar Crops Research and Institute of Field Crops Research, ARC, Giza, Egypt during the 2019 and 2020 seasons.

### 4.1. Plant Materials

The study was carried out on two varieties of *Stevia rebaudiana* Bertoni. The first variety was Sugar High A3, which has lots of branches, larger leaves, is a bushy plant and contains high levels of glycosides, and its seeds were imported from Ever Stevia Company, Canada. The second variety was Spanti, which has small leaves and contains lower levels of glycosides than the Sugar High A3 variety, which originates from Spain; its seeds were imported from Fito Seed Company, Spain. The two varieties were imported for seed production and improvement by tissue culture propagation with single plant selection programs at Sugar Crops Research Institute, Giza, Egypt.

The leaves of the plant were used as the source of explants for callus initiation, regeneration, multiplication, and rooting. After washing with tap water, the leaves were sterilized by immersion in 70% ethanol for 5 min. Leaf sterilization was followed by immersion in the chemical disinfectant Clorox of 20% sodium hypochlorite (NaOCl, 5.25%) that was supplemented with 150 mg L^−1^ of ascorbic acid for 20 min. Then, they were rinsed four times each for ten min in sterile distilled water, containing 150 mg L^−1^ ascorbic acid.

### 4.2. Mode of Excision

Margins of expanded leaves (10–12 mm long) were detached, and the remaining part was cut diagonally to the midrib into 2 portions. Then, the leaf portions were dissected into small pieces (0.3 cm) and positioned with the adaxial surface in touch with Murashige and Skoog (MS) media [81].

### 4.3. Callus Induction, Shoots Regeneration and Rooting

Leaf segments were positioned with the adaxial surface in contact with the MS medium and supplemented with 0.7% agar, 3% sucrose, 0.5 mg L^−1^ indole-3-butyric acid (IBA) 1.0 mg L^−1^ 6-benzyladenine (BA). The pH value of the medium was adjusted to 5.7. Explants were grown using a 16 h light photoperiod with 2000 lux as the light intensity provided by cool white fluorescent tubes at 25 °C. The callus was cultured in MS medium supplemented with 0, 500, 1000, 2000, and 3000 mg L^−1^ NaCl as a source of salinity for in vitro salt stress. The subculture was performed every four weeks using the same medium. Then, the maintained calli were transferred to the same previous MS medium supplemented with 2.0 mg L^−1^ BA and 0.5 mg L^−1^ IBA for the regeneration of shoots. The number of shoots per callus piece was recorded two months after transferring the callus to the regeneration medium. Multiple shoots were separated and transferred vertically on **½** MS medium supplemented with 2% sucrose, 0.7% agar, 0.5 mg L^−1^ IBA and the pH value was adjusted to 5.7 as a rooting medium for 4 weeks. Data were recorded as percentages of survival callus and regeneration of callus, plantlet length (cm), number of multiple shoots, number of leaves per plant, number of roots per plant, and root length (cm).

### 4.4. Estimation of Photosynthetic Pigments and Proline Accumulation

Total chlorophyll concentration measurements were conducted using a spectrophotometer and dimethylformamide extracts of leaf tissue, as described previously by Constan et al. [82]. Leaf tissue for these measurements was harvested using a circular punch (cork borer) that yields 0.5 cm diameter leaf discs that are 0.2 cm^2^ in area; excised discs were also weighed, enabling the chlorophyll data to be expressed to fresh weight as well as leaf area. Each solvent extract contained several leaf discs from multiple different plants per the two stevia varieties sugar High A3 and Spanti under each NaCl concentration. SPAD values were recorded using the same leaves from the same plants, before sampling, using a SPAD-502 m (Konica-Minolta, Japan). SPAD-502 m determines the relative content of chlorophyll by measuring light absorbance at 650 nm (red, maximum absorption of red) and 940 nm (NIR, maximum reflection of near-infrared). Ten independent SPAD measurements were made per sample, using several different plants. Total chlorophyll concentration was estimated as µg cm^−2^. 

The accumulation of proline in leaves was quantified as per the method recommended by Bates et al. [83]. Proline was extracted from 0.5 g fresh leaf samples of stevia with 10 mL of 3% sulfosalicylic acid and then centrifuged at 12,000× *g* for 10 min. A total of 1 mL supernatant was reacted with an equal volume of acid-ninhydrin and glacial acetic acid in a test tube and incubated for 1 h at 100 °C. The reaction was stopped by keeping the test tube in an ice bath. Then, 2 mL toluene was added to the test tube, mixed vigorously, and left undisturbed for 30 min at room temperature. After that, the sample mixture was separated into two phases. The optical density of the chromophore containing toluene was measured at 520 nm with a UV-120 spectrophotometer (Shimadzu Corp., Kyoto, Japan). The proline content was determined based on standard curves developed with D-proline. Proline accumulation was estimated as µmoles g^−1^ of fresh weight leaf material.

### 4.5. Isozymes Electrophoresis

Isozymes electrophoresis was performed in vertical polyacrylamide gels with a discontinuous buffer system, as described by Iglesias and Simon [84]. Isozyme assay: Native-polyacrylamide gel electrophoresis (Native-PAGE) was conducted to identify isozyme variations among 10 samples for two stevia varieties: Sugar High A3 and Spanti, under five NaCl concentrations using the isozyme systems according to Stegemann et al. [85].

For peroxidase (POD), polyphenol oxidase (PPO), and malate dehydrogenase (MDH) analysis, 500 mg fresh leaves were collected in an icebox and homogenized by liquid N_2_ and 100 µL of 0.2 M phosphate buffer (pH 7.0 was adjusted by Potassium Phosphate, monobasic) was added, in addition to 10 µL of 2-Mercaptoethanol, before centrifugation at 14,000 rpm for 15 min at 4 °C to clear the cellular debris. The supernatant was recovered and used directly for electrophoresis or stored at a temperature of −20 °C. All extractions were performed at a temperature of approximately 4 °C and icebox. Peroxidase loci were detected by 0.044 M phosphate-0.028 M citric acid buffer (pH 4.4–4.6) and 0.026% (*w/v*) O-dianisidine and 1% H_2_O_2_ in an incubator at 37 °C temperature until the bands developed sufficiently to permit scoring. The bands were fixed using 7% acetic acid after staining.

Peroxidase isozymes (POD) were visualized by incubating the gels in a solution consisting of 80 mL of 0.2 M acetate buffer (pH 5), 8 mL of 3% H_2_O_2_, 4 mL of 0.04 M benzidine, POD isozymes appeared with brown bands after 30–60 min at 4 °C, according to Rahnama and Ebrahimzadeh [86]. Polyphenol oxidase (PPO) isozymes were detected according to Baaziz et al. [87], in which the gel was immersed in a solution containing 0.1% 1-dihydroxyphenylalanine solubilized in 0.05 phosphate buffer (pH 7.5). Malate dehydrogenase (MDH) was detected according to Jonathan and Wendel [88], in which the gel was soaked in 100 mL of 0.05 M Tris HCl (pH 8.5) containing 25 mg NBT, 25 mg EDTA, 25 mg NAD, 10 mg malic acid and 3 mg PMS. 0.05 M Tris HCl (pH 8.5) was prepared by dissolving 0.605 g Tris in 50 mL distilled water. The pH was adjusted to 8.5 by HCl. Then, the solution was completed to 100 mL by distilled water.

After the appearance of the enzyme bands, the reaction was stopped by washing the gel two or three times with tap water. This was followed by adding the fixative solution, which consists of ethanol and 20% glacial acetic acid (9:11, *v/v*). The gel was kept in the fixative solution for 24 h and then was photographed. All isozyme gels electrophoreses were scanned using a gel documentation system manufactured by Alpha Ease FC (Alphimager 2200), U.S.A. Relative band mobility was measured concerning the dye front and indicated by *Rf* values.

### 4.6. Statistical Analysis

The experiment was set in a completely randomized design. Each treatment was performed in ten jars containing five explants and each experiment was replicated thrice. Data of 50 plantlets per replicate were subjected to analysis. Analysis of variance was used to analyze the experimental data. Duncan’s Multiple Range Test at a 5% level of significance (*p* ≤ 0.05) was used for comparing means according to Snedecor and Cochran [89].

## 5. Conclusions

It can be concluded that the high concentration of NaCl had a negative influence on all the studied variables. Increasing NaCl concentrations were reflected in the decreased shoot number, shoot length, root number, root length, leaf number of stevia in vitro. Finally, a specific advantage of salinity stress is the development of a set of tolerant lines. The results in this study confirmed that Sugar High A3 was more salt-tolerant than the Spanti variety. All variant regenerated plantlets were acclimatized to be transplanted into the permanent field for further studies and utilized in breeding programs and selection procedures.

Some detected biochemical markers peroxidase (PDO), polyphenol oxidase (PPO), and malate dehydrogenase (MDH) were associated with salt tolerance. These markers could be applied in stevia breeding programs, as marker-assisted selection. Therefore, peroxidase, polyphenol oxidase, and malate dehydrogenase enzyme activities and gene expression can be used as biochemical and molecular markers to detect the resistance or susceptibility nature of stevia cultivars against salinity.

## Figures and Tables

**Table 1:** Effects of different NaCl concentrations (0, 500, 1000, 2000, and 3000 mg L^−1^) on callus and plantlets vegetative growth characteristics of stevia varieties (Sugar High A3 and Spanti) in vitro.

Parameters	NaCl mg L^−1^ Concentrations	Stevia Varieties		Means
		Sugar High A3	Spanti	
Survival callus%	Control	62.50 ^a^	62.00 ^a^	62.25 ^a^
	500	53.09 ^b^	50.60 ^b,c^	51.85 ^b^
	1000	52.30 ^b^	43.66 ^d^	47.98 ^c^
	2000	48.05 ^c^	36.17 ^e^	42.11 ^d^
	3000	44.20 ^d^	32.65 ^f^	38.43 ^e^
	Means	52.13 ^a^	45.01 ^b^	
Regeneration%	Control	42.11 ^a^	34.93 ^b,c^	38.52 ^a^
	500	35.83 ^a,b^	30.16 ^c^	32.99 ^b^
	1000	36.03 ^a,b^	23.12 ^d,e^	29.57 ^c^
	2000	35.61 ^b,c^	20.14 ^e^	27.88 ^c,d^
	3000	29.81 ^c^	18.09 ^e^	23.95 ^d^
	Means	35.90 ^a^	25.30 ^b^	
Plantlet length (cm)	Control	5.15 ^b^	4.86 ^b^	5.01 ^a,b^
	500	5.66 ^a^	4.44 ^b^	5.05 ^a^
	1000	4.73 ^b^	4.23 ^b^	4.48 ^a,b^
	2000	4.26 ^b^	3.74 ^b^	4.00 ^b^
	3000	4.02 ^b^	3.13 ^b^	3.58 ^b^
	Means	4.76 ^a^	4.08 ^b^	
No. of leaf plantlets^−1^	Control	9.61 ^a^	8.76 ^ab^	9.19 ^a^
	500	8.83 ^a,b^	7.51 ^a,c^	8.17 ^a,b^
	1000	7.56 ^a,b,c^	7.02 ^b,c,d^	7.29 ^b^
	2000	7.15 ^b,c,d^	5.53 ^d^	6.34 ^c^
	3000	6.11 ^c,d^	5.31 ^d^	5.71 ^c^
	Means	7.85 ^a^	6.82 ^b^	
No. of multiple shoots	Control	15.60 ^a^	12.50 ^b,c,d^	14.05 ^a^
	500	14.42 ^a,b^	10.60 ^d,e,f^	12.51 ^b^
	1000	13.27 ^a,b^	8.36 ^e,f,g^	10.82 ^b,c^
	2000	12.87 ^b,c^	7.68 ^f,g^	10.28 ^c^
	3000	10.23 ^c,d,e^	6.68 ^g^	8.46 ^d^
	Means	13.22 ^a^	9.16 ^b^	
Root length (cm)	Control	7.55 ^a^	6.57 ^c,d^	7.06 ^a^
	500	7.22 ^a,b^	6.23 ^c,d^	6.73 ^a^
	1000	6.71 ^b,c^	6.23 ^d,e^	6.47 ^b^
	2000	6.17 ^c,d,e^	5.21 ^f^	5.49 ^c^
	3000	6.06 ^e^	5.85 ^f^	5.96 ^c^
	Means	6.66 ^a^	6.02 ^b^	
No. of root plantlets^−1^	Control	7.26 ^a^	6.27 ^a,b^	6.77 ^a^
	500	7.31 ^a^	5.05 ^c^	6.18 ^a,b^
	1000	6.38 ^a,b^	5.11 ^c^	5.75 ^b^
	2000	5.26 ^b,c^	3.36 ^d^	4.31 ^c^
	3000	5.01 ^c^	3.76 ^d^	4.39 ^c^
	Means	6.24 ^a^	4.96 ^b^	

Means followed by the same letters in each mean column, mean row, or interaction are not significantly different at the 5% level.

**Table 2:** Densitometric analyses of leaf peroxidase isozyme of stevia varieties (Sugar High A3 and Spanti) grown in different NaCl concentrations (0, 500, 1000, 2000, and 3000 mgL^−1^) in vitro.

Peroxidase Groups	Relative Mobility	Stevia Varieties									
		Sugar High A3					Spanti				
		SuC	Su1	Su2	Su3	Su4	SpC	Sp1	Sp2	Sp3	Sp4
POD1	0.18	0	0	0	0	0	1−	1−	0	0	0
POD2	0.23	0	1−	1−	1−	0	1−	1−	0	0	1−
POD3	0.29	0	0	1−	1−	1−	1−	1−	0	0	1−
POD4	0.34	0	0	1−	1−	0	0	0	0	0	0
POD5	0.52	1+	1−	1++	1++	1++	1+	1++	1+	1++	1++
POD6	0.57	0	0	0	0	1−	0	0	0	0	0
POD7	0.83	1+	0	1++	1++	1++	1−	1++	1++	1++	1++
POD8	0.87	0	0	1−	1−	1−	1−	1+	1−	1−	1−
POD9	0.91	0	0	1−	1−	1+	0	0	0	0	0
POD10	0.98	0	0	0	1−	1−	0	1−	1−	0	1−

SuC: Sugar high A3 variety at control conditions (salt-free); SpC: Spanti variety at control conditions (free salt); Su1: Sugar high A3 at 500 mg L^−1^ NaCl; Sp1: Spanti at 500 mg L^−1^ NaCl; Su2: Sugar high A3 at 1000 mg L^−1^ NaCl; Sp2: Spanti at 1000 mg L^−1^ NaCl; Su3: Sugar high A3 at 2000 mg L^−1^ NaCl; Sp3: Spanti at 2000 mg L^−1^ NaCl; Su4: Sugar high A3 at 3000 mg L^−1^ NaCl; Sp4: Spanti at 3000 mg L^−1^ NaCl; 1 = Present band, 0 = Absent band, ++ = High density band, + = Moderate density band and − = Low density band.

**Table 3:** Densitometric analysis of leaf polyphenol oxidase isozyme of stevia varieties (Sugar High A3 and Spanti) grown in different NaCl concentrations (0, 500, 1000, 2000, and 3000 mgL^−1^) in vitro.

Polyphenol Oxidase Groups	Relative Mobility	Stevia Varieties									
		Sugar High A3					Spanti				
		SuC	Su1	Su2	Su3	Su4	SpC	Sp1	Sp2	Sp3	Sp4
PPO 1	0.38	0	0	0	1−	1−	0	0	0	0	0
PPO 2	0.42	0	0	0	0	−1	0	0	0	0	0
PPO 3	0.58	1++	1+	1+	1++	1++	1+	1+	1+	1+	1+
PPO 4	0.62	0	0	0	1−	1−	0	0	0	0	0
PPO 5	0.85	1++	1−	1−	1++	1++	1+	1+	1−	1++	1−
PPO 6	0.88	1−	0	1−	1−	1−	1−	1−	0	1−	0

SuC: Sugar high A3 variety at control conditions (salt-free); SpC: Spanti variety at control conditions (free salt); Su1: Sugar high A3 at 500 mg L^−1^ NaCl; Sp1: Spanti at 500 mg L^−1^ NaCl; Su2: Sugar high A3 at 1000 mg L^−1^ NaCl; Sp2: Spanti at 1000 mg L^−1^ NaCl; Su3: Sugar high A3 at 2000 mg L^−1^ NaCl; Sp3: Spanti at 2000 mg L^−1^ NaCl; Su4: Sugar high A3 at 3000 mg L^−1^ NaCl; Sp4: Spanti at 3000 mg L^−1^ NaCl; 1 = Present band, 0 = Absent band, ++ = High density band, + = Moderate density band and − = Low density band.

**Table 4:** Densitometric analysis of leaf malate dehydrogenase isozyme of stevia varieties (Sugar High A3 and Spanti) grown in different NaCl concentrations (0, 500, 1000, 2000, and 3000 mg L^−1^) in vitro.

Malate Dehydrogenase Groups	Relative Mobility	Stevia Varieties									
		Sugar High A3					Spanti				
		SuC	Su1	Su2	Su3	Su4	SpC	Sp1	Sp2	Sp3	Sp4
MDH 1	0.29	0	0	0	0	0	0	1−	0	0	0
MDH 2	0.33	1+	1+	1+	1+	1+	1+	1−	0	1−	1−
MDH 3	0.40	0	1−	1−	0	0	0	1−	0	1−	1−
MDH 4	0.52	1+	1+	1+	1+	1+	1+	1+	1−	1+	1+
MDH 5	0.55	1−	1−	0	1−	1−	1−	1−	0	0	0
MDH 6	0.78	1+	1+	1+	1++	1+	1+	1−	1−	1+	1+
MDH 7	0.83	1+	1+	1+	1+	1−	1−	1−	1−	1+	1+

SuC: Sugar high A3 variety at control conditions (salt-free); SpC: Spanti variety at control conditions (free salt); Su1: Sugar high A3 at 500 mg L^−1^ NaCl; Sp1: Spanti at 500 mg L^−1^ NaCl; Su2: Sugar high A3 at 1000 mg L^−1^ NaCl; Sp2: Spanti at 1000 mg L^−1^ NaCl; Su3: Sugar high A3 at 2000 mg L^−1^ NaCl; Sp3: Spanti at 2000 mg L^−1^ NaCl; Su4: Sugar high A3 at 3000 mg L^−1^ NaCl; Sp4: Spanti at 3000 mg L^−1^ NaCl; 1 = Present band, 0 = Absent band, ++ = High density band, + = Moderate density band and − = Low density band.

## Data Availability

The data presented in this study are available upon request from the corresponding author.
